# Summary and Analysis of Digital Pain Manikin Data in Adults With Pain Experience: Scoping Review

**DOI:** 10.2196/69360

**Published:** 2025-08-22

**Authors:** Darcy Alex Murphy, Syed Mustafa Ali, Shellie Ann Boudreau, William Dixon, David Wong, Sabine N van der Veer

**Affiliations:** 1 Division of Informatics, Imaging and Data Sciences University of Manchester Manchester United Kingdom; 2 Content Avenue Aalborg Denmark; 3 Institute of Health Informatics University of Leeds Leeds United Kingdom

**Keywords:** digital pain manikin, digital pain drawing, digital pain body map, digital pain chart, pain measurement, patient-generated health data, artificial intelligence, AI

## Abstract

**Background:**

A digital pain manikin is a measurement tool that presents a diagram of the human body where people mark the location of their pain to produce a pain drawing. Digital pain manikins facilitate collection of more detailed spatial pain data compared to questionnaire-based methods and are an increasingly common method for self-reporting and communicating pain. An overview of how digital pain drawings, collected through digital pain manikins, are analyzed and summarized is currently missing.

**Objective:**

This study aimed to map the ways in which digital pain drawings were summarized and analyzed and which pain constructs these summaries attempted to measure. The objectives were to (1) identify and characterize studies that used digital pain manikins for data collection, (2) identify which individual drawing–level summary measures they reported and the methods by which these summaries were calculated, and (3) identify if and how multidrawing (eg, time series) summary and analysis methods were applied.

**Methods:**

We conducted a scoping review to systematically identify studies that used digital pain manikins for data collection and reported summary measures or analysis of the resulting digital pain drawings. We searched multiple databases using search terms related to *pain* and *manikin*. Two authors independently performed title, abstract, and full-text screening. We extracted and synthesized data on how studies summarized and analyzed digital manikin pain data at the individual pain–drawing level as well as across multiple pain drawings.

**Results:**

Our search yielded 6189 studies, of which we included 92. The majority were clinical studies (n=51) and cross-sectional (n=64). Eighty-seven studies reported at least 1 individual drawing–level summary measure. We identified individual drawing–level manikin summary measures related to 10 distinct pain constructs, with the most common being pain extent (n=53), physical location (n=28), and widespreadness (n=21), with substantial methodological variation within constructs. Forty-two studies reported at least 1 multidrawing summary method. Heat maps were most common (n=35), followed by the number or proportion of participants reporting pain in a specific location (n=14). Sixteen studies reported multidrawing analysis methods, the most common being an assessment of the similarity between pairs of pain drawings representing the same individual at the same moment in time (n=6).

**Conclusions:**

We found a substantial number of studies that reported manikin summary and analysis methods, with the majority being cross-sectional clinical studies. Studies commonly reported pain extent at the individual–drawing level and used heat maps to summarize data across multiple drawings. Analysis methods that went beyond summarizing pain drawings were much rarer, and methodological variation within pain constructs meant a lack of comparability between studies and across manikins. This highlights a need for development of standardized methods that are applicable across manikins and more advanced methods that harness the spatial nature of pain drawings.

## Introduction

### Background

It is necessary to measure pain for a variety of clinical and research purposes, including etiology, diagnosis, monitoring disease state, and measuring and understanding intervention effect. Pain measures are part of the diagnostic criteria and classification criteria for various conditions, including fibromyalgia [1], chronic migraine [2], osteoarthritis [3], and rheumatoid arthritis [4]. Pain is also a common symptom for cancer, and the location of the pain is associated with the type and stage of the cancer [5].

Digital pain manikins, also known as pain drawings, pain charts, or pain body maps, are an increasingly common tool used to gather self-report pain data. They are an outline diagram of a human body, typically with a front and back view. Newer digital versions may provide more detail by using shading to indicate the breasts and chest cavity or the knee shape and structure [6]. People self-report pain by marking or coloring the location of their pain using a touch screen or mouse [7]. A key feature of pain manikins compared to other pain instruments is that people can self-report pain spatially. This gives pain manikins unique potential as a pain measurement tool. Throughout this review, we use the term “digital pain manikin” to refer to the tool and “pain drawing” to refer to an instance of a report created using a digital pain manikin.

We categorized the summary and analysis of digital pain drawings as an individual drawing–level summary measure, a multidrawing summary method, or a multidrawing analysis method. Individual drawing–level summary measures quantify an aspect of an individual’s pain experience at a specific moment in time. For example, pain extent (also referred to as pain area) quantifies the area of pain as marked on a single pain drawing [8]. Multidrawing summary methods give information about pain across a population, across time, or both. For example, heat maps (images visualizing an average of multiple pain drawings) can show the most common locations for an individual’s pain over time or the average pain profile for a specific condition across a population [9]. Multidrawing analysis methods provide direct interpretation of digital pain drawings rather than only compressing them, for example, by using machine learning clustering methods to group similar pain drawings together and characterize the distribution of pain [10].

Previous systematic reviews have noted a lack of standardization both in pain manikins and in summary measures derived from them [7,11], which may introduce problems with reproducibility of results. It also limits the ability to compare and synthesize results meaningfully across studies. For example, drawing from measurement theory [12], a lack of comparable conceptualizations of the constructs that are to be measured hampers the assessment of measurement properties (such as reliability and validity) of manikin-derived summary measures. Understanding the current state of how digital pain drawings derived from digital manikins are summarized and analyzed in the field is, therefore, a crucial step toward building more robust, reproducible, and scalable methods.

A 2019 systematic review of methodological milestones in pain manikins divided manikin-derived measures into “topographic” and “simple” measures, with topographic measures being those incorporating anatomical knowledge [7]. They found the most common simple measures to be those quantifying the size of the painful area and widespreadness to be the most widely used topographic measure. However, mapping digital pain manikin summary measures and analysis methods was not a focus of the review. This means that the full picture of which manikin-derived summary measures and analysis methods are being used and which pain constructs these relate to is not currently established [7].

Therefore, this review maps the ways digital pain drawings are summarized and analyzed, including the pain constructs measured using digital pain manikins.

### Objectives

The specific objectives were to (1) identify and characterize studies that used digital pain manikins for data collection, (2) identify (a) which individual drawing–level summary measures they reported and (b) the methods by which these summaries were calculated, and (3) identify if and how multidrawing (eg, time series) summary and analysis methods were applied.

We expect this review to inform the direction of future work on developing more advanced manikin-derived summary measures and analysis methods that make best use of the spatial information manikins provide. Ultimately, this will contribute to harnessing the potential of digital manikins to support pain outcome measurement in both research and clinical care.

## Methods

### Overview

We reported this review in line with the PRISMA (Preferred Reporting Items for Systematic Reviews and Meta-Analyses) reporting guidelines for scoping reviews [13]. The completed PRISMA-ScR (Preferred Reporting Items for Systematic Reviews and Meta-Analyses Extension for Scoping Reviews) checklist is available in Multimedia Appendix 1.

We limited the scope of this review to digital pain manikins, excluding paper pain manikins. The main reasons were that the field is shifting away from paper manikins and toward digital manikins [7] and that we anticipated that any summary or analysis methods applied to paper manikins were likely to also be represented in studies using digital manikins.

### Information Sources and Search

We used the same search strategy as for a related review, which was registered on PROSPERO (an international database of prospectively registered systematic reviews in health and social care) [14].

We searched MEDLINE, CINAHL and Embase via Ovid, Scopus, IEEE Xplore, and the ACM Digital Library using search terms related to *pain* and *manikin*, including a range of common synonyms such as *pain drawing* and *pain body chart*. The full search strategy is included in Multimedia Appendix 2. The search was not restricted based on publication date and was complemented by hand searching reference lists of included studies. We did not search additional sources for gray literature. We originally ran the search in November 2019 and updated the search in August 2023. The search strategy was developed by researchers with experience of conducting systematic reviews and supported by a qualified librarian.

### Eligibility Criteria

Papers were included if they were published in English and met the criteria outlined in Textbox 1.

**Eligibility criteria.**

- Study population: adults aged >16 years, including people with pain or a painful condition and healthy volunteers. Studies with a mixed sample of adults and children were included.
- Digital pain manikin: studies that used a digital pain manikin for data collection, defined as any human-shaped figure that facilitated interactive self-reporting of pain in any part or location of the body on a digital device, for example, a desktop computer, tablet, smartphone, or custom device. Manikins focusing on a specific body part were included.
- Intended manikin users: adults with current or previous personal pain experience. In other words, studies were included if manikins were intended to be used for self-reporting pain by the person who experienced the pain. This included healthy volunteers reporting induced pain. Studies that consisted solely of pain drawings created by health care professionals or researchers to record their observations of patients’ pain were excluded, but those with manikins completed by both patients and others were included.
- Outcome of interest: we included studies that reported any summary of the data collected using digital pain manikins. This included methods for summarizing the information from a single pain drawing (eg, pain extent) and for aggregating information across pain drawings (eg, a heat map showing where study participants most commonly reported pain). We included summaries that were calculated automatically (eg, pain extent extracted automatically by the manikin software) and those that were generated manually (eg, a visual assessment of pain symmetry).
- Publication type: original research, including peer-reviewed journals and full conference papers, excluding gray literature, preprints, protocols, reviews, commentaries, editorials, and conference abstracts.

### Selection of Sources of Evidence

After deduplication, we performed title and abstract screening to identify potentially relevant papers, followed by full-text screening to confirm eligibility. Deduplication was performed by a single author (DAM). For both screening stages, all papers were screened independently by pairs of reviewers (SMA, DAM, Dr Rebecca Lee and Danielle Mountain), with disagreements resolved by discussion with a third author (SvdV).

### Data Charting Process

We recorded whether a study was a clinical study, where a manikin was used for data collection to answer a clinical research question, or a development or validation study, where the primary aim of the study was one or both of the development and testing of a digital pain manikin. We also recorded whether a manikin was 2D, 3D, or pseudo-3D. We defined a 3D manikin to be a manikin with a rotatable model, as opposed to a pseudo-3D manikin with a fixed 2D perspective but with additional visual detail and shading that gave it a 3D appearance.

We developed a data charting form and pilot-tested it on 10 papers before starting full data charting. Data charting was performed for all included papers by one author (DAM), with 25% (23/92) in duplicate by a second author (SMA). Missing data on study, setting and population, and manikin characteristics was noted as “not reported” during data charting or extracted from references to previous studies using the same manikin or dataset.

### Data Items

We extracted data items related to study characteristics, setting and population characteristics, manikin characteristics, individual drawing–level summary measures and the methods used to produce them, multidrawing summary measures (ie, cross-sectional pain drawings across multiple individuals, multiple pain drawings of an individual over time, or multiple pain drawings of multiple individuals over time), and other analysis methods. Data items on manikin characteristics included which location-specific pain aspects could be recorded on the manikin, including location-specific pain quality (eg, burning or tingling) and location-specific intensity (typically on a scale of 1-10). This is distinct from additional nonmanikin measures collected at the same time (eg, an overall pain intensity score or participant ratings of the usability of the manikin). The full list of data extraction items is available in Multimedia Appendix 3.

*Individual drawing–level manikin summary measures* were defined as any variable extracted directly from a single pain drawing, compressing the high-dimensional pain drawing data into a single measurement, such as pain extent.

*Multidrawing summary methods* were defined as any method of combining (or compressing) data from multiple individual pain drawings without first summarizing the individual manikins, such as heat maps showing the average of multiple pain drawings.

*Multidrawing analysis methods* were defined as a method that produced new information about the data (eg, the use of principal component analysis to assess the knee pain distribution), as opposed to multidrawing summary methods that only compressed the data.

When a study reported descriptive summary statistics of individual summary measures, we extracted this as an individual drawing–level summary measure and not a multidrawing summary method. For example, if a study calculated the pain extent for each individual pain drawing and then reported the average pain extent across participants at baseline and follow-up, we recorded pain extent as an individual drawing–level manikin summary measure and did not record mean pain extent as a multidrawing summary measure.

We defined automated measures as those that were extracted without human intervention on an individual drawing level. For example, if the calculation of pain extent required manual tracing of the pain area, this was counted as manual even if part of the process was performed automatically. If a measure was not explicitly stated to be manual or automated but we could derive it from contextual information, we recorded this as manual or automated (assumed). For example, in studies involving thousands of manikins, manual processing was unlikely, so measures were assumed to be automated unless there was evidence to the contrary.

### Synthesis of Results

Guided by our objectives, we performed a narrative synthesis of the extracted data. For the synthesis of individual drawing–level summary measures (objective 2), we named and defined pain constructs after performing data extraction. A construct is an abstract concept that cannot be directly measured.

We defined pain constructs descriptively according to the following principles:

- Each construct should be defined such that it did not overlap with any of the other constructs, allowing each summary measure to be sorted into only one construct.
- There should be one construct for each summary measure, so that no summary measures were left without a construct to be sorted into.
- Each construct should be defined based on the underlying theoretical construct that was being measured rather than the method used to measure it.

We recorded unique individual drawing–level summary measures within constructs when there were significant methodological variations in how that construct was measured (eg, pain extent with region-based and pixel-based measures) or where there were minor conceptual variations within the construct (eg, pain presence or absence inside a specific anatomical location versus pain presence or absence outside a specific anatomical location). The level of conceptual variation at which we defined a new construct instead of a summary measure within a construct was a subjective distinction. It would, for example, also be possible to consider pain presence inside versus outside a specific anatomical location as separate constructs, rather than different measures within the same construct.

## Results

### Overview

**Figure 1:**
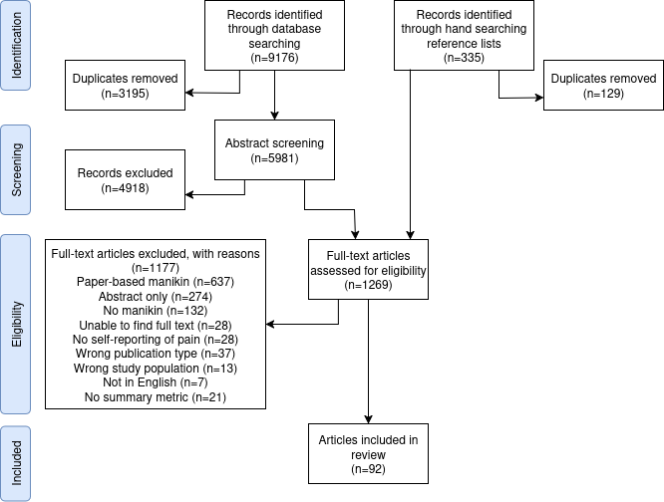
PRISMA (Preferred Reporting Items for Systematic Reviews and Meta-Analyses) diagram showing the screening process with the number of papers excluded at each stage.

Figure 1 shows that our search identified 5981 papers after deduplication, with another 208 identified via our hand search, resulting in a set of 6189 papers to be screened. Finally, we included 92 papers. The main reasons for excluding full papers were that they used paper-based manikins (637/6189, 10.29%) or did not use a manikin at all (132/6189, 2.13%). Of the 92 included studies, 87 (95%) reported at least 1 individual drawing–level summary measure, 42 (46%) reported at least 1 multidrawing summary, and 16 (17%) reported direct analysis of multiple pain drawings.

### Study and Manikin Characteristics

Table 1 shows the characteristics of the included studies. Most studies were conducted in the United States (31/92, 34%) or Denmark (18/92, 20%), were clinical studies (51/92, 55%), and collected data cross-sectionally (64/92, 70%).

Across all studies, we identified 27 unique, named manikins. Almost a quarter of included studies used the Navigate Pain manikin (21/92, 23%), and 23 (25%) studies did not report details on which manikin they used. Most studies used a 2D (61/92, 66%) or pseudo-3D (21/92, 23%) manikin. A total of 3 studies reported using a 3D manikin and 2 compared manikins of different dimensions; these were all developmental or validation studies. Manikins were used across a variety of conditions, primarily for chronic pain (52/92, 57%).

**Figure 2:**
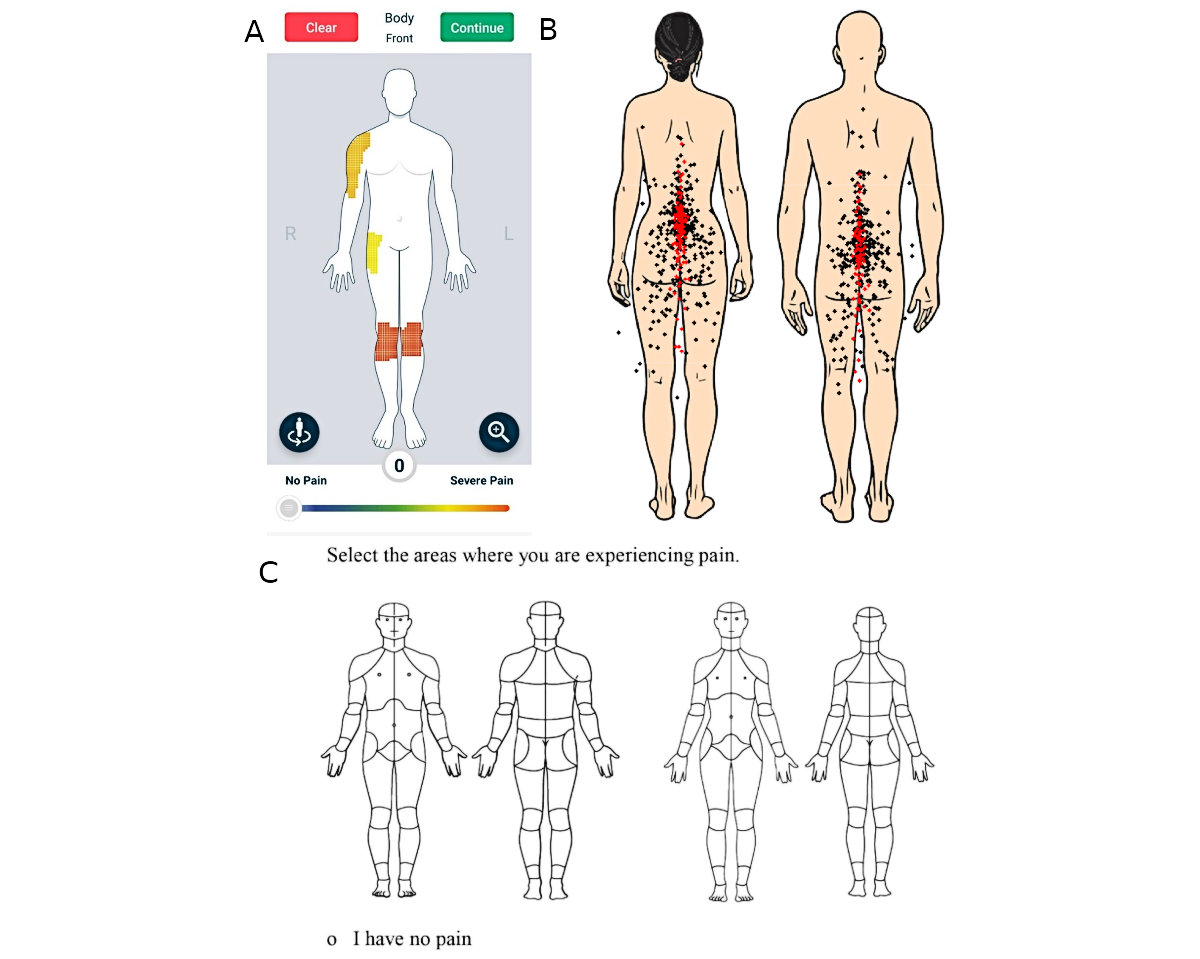
(A) A screenshot of the Manchester Digital Pain Manikin app (uMotif Limited), where users marked the location and intensity of pain on a grid (as described in the study by Ali et al [21]); (B) the manikin used in the study by Miękisiak et al [20], where users made individual marks indicating pain location; and (C) the Collaborative Health Outcomes Information Registry body map, from the study by Scherrer et al [22], where users marked the location and intensity of pain on predefined body regions.

Manikins were most commonly either pixel-based manikins where participants could draw freely (56/92, 61%), analogous to paper-based manikins (such as the Navigate Pain manikin [15]), or manikins with nonoverlapping predefined regions that participants can select (21/92, 23%; eg, the CHOIR (Collaborative Health Outcomes Information Registry) manikin [16]). Exceptions to this include the Manchester Digital Pain Manikin, which used a grid [17]; the manikin used by Zuhdi et al [18] with overlapping predefined joints and areas; an early iteration of the Iconic Pain Assessment Tool, which uses drag-and-drop icons [19]; and the manikin used by Miękisiak et al [20], where users clicked with a mouse to make individual marks rather than shading areas. Example manikin images are shown in Figure 2 [20-22].

**Table 1:** Summary of the characteristics of studies included in the review, including characteristics of the manikins used by those studies (N=92).

Characteristics				Studies, n (%)
**Study characteristics**				
	**Country**			
		United States	31 (34)	
		Denmark	18 (20)	
		Spain	7 (8)	
		United Kingdom^a^	6 (7)	
		France	5 (5)	
		Germany	5 (5)	
		Canada	4 (4)	
		Poland	4 (4)	
		Other^b^	12 (13)	
	**Condition**			
		Chronic pain	52 (57)	
		Musculoskeletal pain	16 (17)	
		Acute pain	9 (10)	
		Neurological pain	6 (7)	
		Dental or facial pain	4 (4)	
		Not reported	5 (5)	
	**Study type**			
		Clinical	51 (55)	
		Development or validation	32 (35)	
		Both	9 (10)	
	**Study period**			
		Cross-sectional	64 (70)	
		Longitudinal	26 (28)	
		Not reported	2 (2)	
**Manikin characteristics**				
	**Detail level**			
		Pixels	56 (61)	
		Predefined regions	21 (23)	
		Grid	4 (4)	
		Squares^c^	3 (3)	
		Scalable vector graphics^d^	3 (3)	
		Circles^e^	1 (1)	
		Icons	1 (1)	
		Not reported	3 (3)	
	**Dimensions**			
		2D only	61 (66)	
		Pseudo-3D only	21 (23)	
		3D only	3 (3)	
		Multiple	2 (2)	
		Not reported	5 (5)	
	**Location-specific pain aspects^f^**			
		None	59 (64)	
		Intensity	20 (22)	
		Quality	15 (16)	
		Depth^g^	4 (4)	
		Other	5 (5)	
		Not reported	3 (3)	

^a^One study was conducted in both Switzerland and the United Kingdom.

^b^Other countries represented included Australia, Belgium, Greece, Italy, Japan, Lebanon, Norway, Switzerland, and Thailand.

^c^Squares were added in the locations that the patient clicked and were not aligned to a grid.

^d^A method of storing image data that records individual markings and their spatial relationship to each other rather than recording the values of individual pixels.

^e^Patients marked the location of worst pain with a small circle.

^f^Eight studies had multiple location-specific pain aspects, so these numbers do not add up to 100%.

^g^How deep into the body the pain was, for example, surface level or in the muscle.

### Individual Manikin Summary Measures

#### How Studies Summarized Data From an Individual Manikin Report

Table 2 lists the 31 unique individual drawing–level summary measures we identified and mapped to 10 pain constructs. The construct definitions are included in Multimedia Appendix 4. A total of 5 summary measures lacked sufficient information to classify them into a specific construct [23-27]. The measures could be split into spatial measures and nonspatial measures. We defined spatial measures to be those that used the physical location of the pain in some way and nonspatial measures to be those that discarded location-specific information. Almost all the measures were spatial measures, including for assessing the size and shape of the painful area or the spread of pain throughout the body. Only 9 studies reported nonspatial measures, all of which were pain quality measures, which summarized the presence or number of pain quality descriptors or the maximum or minimum pain intensity marked anywhere on the drawing.

*Pain extent* (also called pain area) was the most widely measured construct, with 53 of the 87 studies reporting related individual summary measures. The main methodological variation in assessing and reporting pain extent related to the granularity of the manikin data, as pain extent for pixel-based manikins was generally reported as the percentage or raw number of pixels, whereas for manikins using predefined areas, this was generally calculated by weighting the size of each marked pain area. In comparison, there were no clear differences between pixel- and region-based manikins in calculating pain location measures (n=28), despite the difference in the level of detail available.

**Table 2:** Individual summary measures reported by the included studies, grouped by pain construct, along with the corresponding studies that reported each measure (N=87).

Description (number of studies; number of reported measures calculated automatically, manually, and not reported^a^)			Studies
**Pain extent (n=53, 61%; n=51, 59% automated, n=5, 6% manual, and n=3, 3% not reported)**			
	Pain area in absolute number of pixels (n=24, 28%)	[8-10,15,23,24,28-44]	
	Pain area as a percentage of marked pixels (n=13, 15%)	[6,15,25,35,45-53]	
	Pain area quantified using predefined anatomical regions (n=5, 6%)	[8,28,54-56]	
	Pain area quantified without the use of pixels or predefined regions (n=6, 7%)	[8,17,57-60]	
	Pain area quantified as the physical area (n=2, 2%)	[61,62]	
	Pain area for specific symptoms (n=3, 3%)	[32,63,64]	
	Unspecified (n=6, 7%)	[20,26,65-68]	
**Location (n=28, 32%; n=14, 16% automated, n=9, 10% manual, and n=7, 8% not reported)**			
	Presence or absence in a specific anatomical location (n=5, 6%)	[10,16,27,30,69]	
	Presence or absence outside a specific anatomical location (n=5, 6%)	[16,70-73]	
	Description of the pain location (n=5, 6%)	[57,74-77]	
	Which predefined areas have pain presence (n=5, 6%)	[9,22,38,55,78]	
	The area of pain in a specific location or locations (n=9, 10%)	[35,73,79-85]	
	Unspecified (n=1, 1%)	[86]	
**Widespreadness (n=21, 24%; n=9, 10% automated, n=4, 5% manual, and n=13, 15% not reported)**			
	Widespread pain index (n=3, 3%)	[25,53,87]	
	Clinical or categorical definitions (n=5, 6%)	[23,77,88-90]	
	Number of predefined areas marked as painful (unspecified number of predefined areas included in manikin; n=9, 10%)	[9,22,23,70,75,77,86,91,92]	
	Number of predefined areas marked as painful (15 or fewer predefined areas included in manikin; n=3, 3%)	[88,93,94]	
	Number of predefined areas marked as painful (16 to 69 predefined areas included in manikin; n=2, 2%)	[8,90]	
	Number of predefined areas marked as painful (70 or more predefined areas included in manikin; n=4, 5%)	[88,95-97]	
**Pain quality (n=9, 10%; n=5, 6% automated, n=1, 1% manual, and n=5, 6% not reported)**			
	Presence or absence of a particular pain quality (n=5, 6%)	[19,39,45,63,98]	
	The number of pain quality or symptom descriptors used (n=2, 2%)	[25,96]	
	Maximum intensity reported anywhere on the drawing (n=3, 3%)	[57,80,96]	
	Minimum intensity reported anywhere on the drawing (n=1, 1%)	[96]	
**Laterality (n=7, 8%; n=2, 2% automated, n=4, 5% manual, and n=1, 1% not reported)**			
	Whether pain is present on one or both sides of the body split vertically (n=7, 8%)	[10,30,35,39,45,91,99]	
**Symmetry (n=5, 6%; n=2, 2% automated, n=3, 3% manual, and n=1, 1% not reported)**			
	The degree to which pain is mirrored on the vertical midline of the body (n=5, 6%)	[10,20,30,39,46]	
**Shape (n=5, 6%; n=4, 5% automated, and n=3, 3% not reported)**			
	The length of the area of pain (n=4, 5%)	[37,68,73,74]	
	The width of the area of pain (n=1, 1%)	[68]	
	The product of the maximum width and length of the area of pain (n=2, 2%)	[50,74]	
**Location-specific intensity (n=4, 5%; n=4, 5% automated, n=1, 1% manual, and n=1, 1% not reported)**			
	Weighted score for pain intensity using location-specific pain intensity information (n=4, 5%)	[52,62,80,96]	
**Overlap (n=3, 3%; n=2, 2 automated, and n=1, 1% manual)**			
	The area of intersection of 2 distinct co-occurring sensations (n=3, 3%)	[26,61,65]	
**Mismatch (n=3, 3%; n=2, 2 automated, and n=1, 1% manual)**			
	The area of nonintersection of 2 distinct co-occurring sensations (n=3, 3%)	[26,29,65]	

^a^Individual summary measure counts may add up to more than the number of studies that reported the overarching construct due to some studies reporting the same construct measured using multiple methods.

*Location* measures were reported by 28 included studies, quantifying where the reported pain was physically located. Most commonly, this was a binary variable quantifying whether pain was present inside (n=5) or outside (n=5) a predefined anatomical region. These measures were typically associated with conditions characterized by pain in a specific location, such as interstitial cystitis/bladder pain syndrome [72].

*Widespreadness* measures were reported by 21 included studies. The main variations within these measures were whether they were reported as categorical (eg, widespread or not widespread) or as a count of the number of painful areas, whether or not additional criteria beyond the number of painful areas were required (eg, 4 painful areas within one arm would not count, whereas a total of 4 painful areas distributed between the arms and back would), and the number of predefined areas the manikin was divided into (eg, a manikin may be split into 7 predefined areas or 70). There was some overlap in the use of the constructs of pain extent and widespreadness, and it was not always clear which of the two constructs a study intended to measure.

*Pain quality* (n=9) and *location-specific intensity* (n=4) measures were reported by 12 studies, despite 33 studies collecting data on location-specific pain aspects (such as location-specific pain intensity). Pain quality and location-specific intensity were the only constructs that used location-specific pain aspects. This means that 21 studies collected location-specific pain aspects and did not use them as part of individual drawing–level summary measures.

*Laterality* was reported by 7 studies and *symmetry* by 5 studies; only 1 study reported symmetry without also reporting laterality. Similarly, *overlap* (n=3) and *mismatch* (n=3) were both reported by 2 of the 3 studies reporting each of them.

#### Methods Used to Calculate Individual Manikin Summaries

There was considerable methodological variation within similar summary measures. For example, we found 4 different approaches to measuring symmetry within the 5 papers that reported this measure [10,20,30,39,46], ranging from manual expert assessment to an automated algorithm comparing the pain extent on the left and right halves of the body. A total of 4 studies used location-specific pain intensity information to calculate a weighted score that combined pain extent with intensity [52,62,80,96]. The types of manikins and methods used to calculate the measures were different for each study.

Within the measures of pain extent, the most common method was calculating the number of pixels marked as painful, either as an absolute number or as a percentage (n=36). The methods that did not use pixels or predefined regions included calculating the area of the polygon with the smallest number of sides that could enclose each stroke of a scalable vector graphics image [8] or calculating the proportion of available squares shaded with pain intensity >1 [17]. Pain extent measures derived from 3D manikins reported the total number of marks made on the diagram [57], the percentage of the surface area marked as painful [80], or the surface area marked as painful based on the number of predefined regions selected [56].

#### Automation of Manikin Summary Measures

Overall, we found that mostly simpler summary measures were calculated using automated methods. For example, of the 53 studies reporting pain extent, 46 reported (assumed) automated measures (Table 2). In contrast, 3 of the 5 studies used a manual method for symmetry measures. An example of an automated summary measure that made good use of the spatial information available was the symmetry measure developed by Boudreau et al [30], which involved mirroring a pain drawing from 1 knee and translating the mirrored image on the opposite knee to the location with maximum overlap. This avoided the potential problem of an automated symmetry measure giving a low symmetry score to a pain drawing that a human expert would assess as symmetrical due to minor differences in the location of the pain areas. Many studies (n=50) had at least 1 reported measure that was not clearly stated to be manual or automated.

### Multidrawing Summary Methods

**Figure 3:**
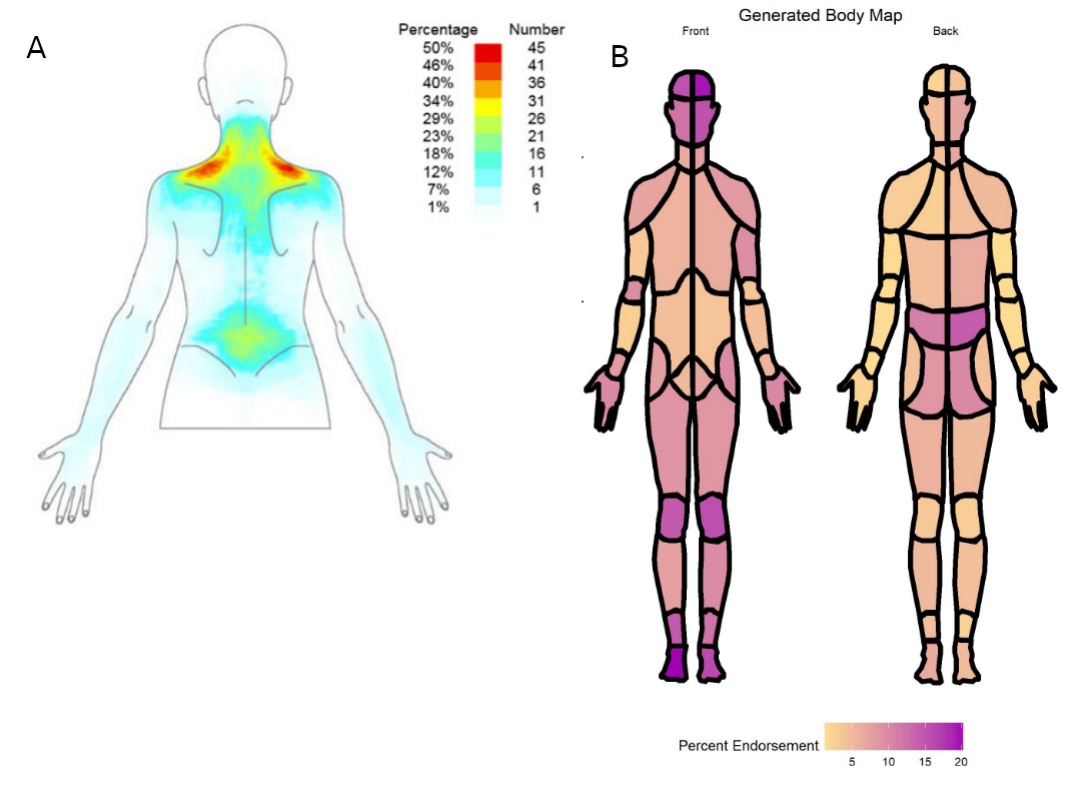
Heat maps reproduced as examples of different digital pain manikin heat maps: (A) Cruder et al [9] generated a pixel average of overlaid pain drawings and (B) Cramer et al [100] generated a region-based average of overlaid pain drawings.

Table 3 shows the 5 multidrawing summary methods we identified. Of the 42 papers summarizing data from multiple pain drawings, 38 reported cross-sectional summaries of populations, 2 reported a summary of 1 individual over time, and 3 reported summaries of populations over time. A total of 4 studies incorporated location-specific pain aspects in their multidrawing summary methods: 2 used site- and symptom-specific methods and 2 used maximum symptom methods. A total of 35 studies presented a heat map. This included pixel-wise averages of multiple individual manikins (n=23; Figure 3 [9,100]); the region-based equivalent showing the proportion of reports selecting each individual region (n=7); and pixel-wise averages with some type of additional processing, such as a minimum threshold for the number of participants reporting pain in a particular location (n=4). Most heat maps (n=32) were cross-sectional summaries of populations.

Studies that reported the number of participants with pain in a particular location (n=14) for pixel-based manikins (n=9) calculated this by overlaying predefined regions and counting the number of participants who marked pain within each region; the other 5 studies reporting this measure used region-based manikins. A total of 2 studies reported site- and symptom-specific summaries; one study provided the number of participants who reported a specific pain quality at a specific site (eg, throbbing and pulsing) [98], while the other study reported the average Numeric Rating Scale score for specific symptoms (frequency of interference, intensity, and influence on playing) at a specific site [18].

**Table 3:** The methods used to summarize multiple pain drawings (ie, multidrawing summary methods). Heat map summaries are split into multiple rows to capture the variation in methods (N=42).

Name and general definitions (number of studies)		Population, time period, or both^a^	Studies
**Heat map (n=35, 83%)**			
	Simple pixel average of overlaid pain drawings (includes Scalable Vector Graphics; n=21, 50%)	Population	[9,10,29,30,33,34,36-38,40,45,48-51,54,67,73,82,101]
	Simple pixel average of overlaid pain drawings at different points in time (n=2, 5%)	Both	[41,74]
	Not described (pixel average of pain drawings from multiple players over time; n=1, 2%)	Not reported (both)	[64]
	Simple region-based average of overlaid pain drawings, with or without a histogram (n=7, 17%)	Population	[16,28,38,51,84,89,95,100]
	Pixel average of overlaid pain drawings with additional processing, such as mirroring or a minimum threshold for number of participants reporting pain in that location (n=4, 10%)	Population	[25,39,46,66]
	Unclear (n=2, 5%)	Population	[53,102]
**Location frequency (n=14, 33%)**			
	The number or percentage of participants reporting pain in specific predefined locations (n=14, 33%)	Population	[9,18,27,28,38,40,46,54,66,79,82,84,90,91]
**Site and symptom specific (n=2, 5%)**			
	The number of participants reporting specific symptoms in specific locations (n=1, 2%)	Population	[98]
	Average symptom at the specific body site (n=1, 2%)	Population	[18]
**Maximum symptom (n=2, 5%)**			
	Highest value for a specific symptom over a period (n=2, 5%)	Time	[74,93]
**Variation over time (n=1, 2%)**			
	Daily range in number of sites reported (n=1, 2%)	Time	[93]

^a^Whether methods summarized a cross section of a population (“population”), an individual over “time period”, or a population over time (“both”).

### Multidrawing Analysis Methods

The most common analysis performed directly on pain drawings was an assessment of similarity between linked pairs of pain drawings, including pairs generated by researchers copying an example drawing [17] and patient-clinician pairs where the clinician completed a pain drawing based on the patient’s verbal description of their pain [50] (n=6), as shown in Table 4. Of the 6 studies that assessed similarity, 3 calculated the Jaccard index, 2 counted the number of pixels colored in both pairs, and 1 performed manual assessment. Other studies used the Jaccard index as a measure of similarity as part of other analysis methods, for example, Galve Villa et al [42] used it to assess change over time and Alter et al [95] used it in a machine learning clustering technique to identify subgroups in a population. Van der Veer et al [17] used the Jaccard index as part of their assessment of test-retest reliability.

Of the 15 papers reporting a multidrawing analysis method, 9 were developmental or validation papers, 5 were clinical, and 1 was both clinical and developmental or validation. All similarity analysis methods (n=6) were part of development or validation studies for the purpose of evaluating the validity of the derived scores.

Studies also used multidrawing analysis methods to assess change over time (n=3), cluster similar drawings together (n=2), categorize drawings by diagnosis (n=2), or correlate pain location information with other data (n=2). Clustering is a machine learning technique for grouping similar examples together and requires a measure of similarity to be successful. Of the clustering studies, the study by Boudreau et al [10] used principal component analysis for dimensionality reduction followed by k-means clustering, and the study by Alter et al [95] performed hierarchical clustering using the Jaccard index as a measure of similarity. The study by Boudreau et al [10] was the only included study that characterized different patterns of pain distribution within an otherwise homogenous diagnosis, in comparison to other studies that simply summarized pain extent or widespreadness. Preserving the spatial information allowed them to identify 3 subgroups within patellofemoral pain that would not have otherwise been distinguishable. Of the studies that analyzed change over time, 1 study investigated the difference between consecutive pairs of pain drawings [42], and 2 studies reported the area under the pain area-time curve, to quantify the change in pain extent over time [68,74].

Ellingsen et al [45] performed pixel-wise correlation with a pain catastrophizing score, which they defined as “a pain-targeted psychosocial construct comprised of helplessness, pessimism, and magnification of pain-related symptoms and complaints.”

**Table 4:** The methods used to analyze reports from multiple pain drawings (ie, multidrawing analysis methods; N=15)^a^.

Type of analysis and methods (number of studies)		Population, time period, both, or pairs^b^	Clinical or developmental	Studies
**Similarity (n=6** **, 40%** **)**				
	Manual assessment of similarity (n=1, 67%)	Pairs	Clinical	[75]
	Number of pixels colored in both of a pair of body maps (ie, pain drawings) (n=2, 13%)	Pairs	Clinical	[29,33]
	Jaccard index (n=3, 50%)	Pairs	Clinical	[17,25,50]
**Change over time (n=3, 20%)** ^c^				
	Area under the pain area-time curve (n=2, 13%)	Time	Clinical	[68,74]
	Jaccard index calculated between consecutive pairs of pain drawings (n=1, 7%)	Time	Both	[42]
**Clustering (n=2, 13%)**				
	Principal component analysis and k-means clustering (n=1, 7%)	Population	Clinical	[10]
	Hierarchical clustering using the Jaccard index as the similarity measure (n=1, 7%)	Population	Clinical	[95]
**Categorization (n=2, 13%)**				
	Simple decision model categorizing drawings into “patient” or “healthy” based on the number of marks made (n=1, 7%)	Population	Developmental	[59]
	Discriminant analysis^d^ based on the proportion of area marked in specific regions (n=1, 7%)	Population	Developmental	[83]
**Location correlation (n=2, 13%)**				
	Pixel-wise correlation with the pain catastrophizing score (presented as heat map; n=1, 7%)	Population	Clinical	[45]
	Intergroup comparisons using *z* tests on a categorical location variable (n=1, 7%)	Population	Developmental	[100]

^a^Excluding studies that first calculated individual-level summary measures (eg, pain extent) and then reported a descriptive summary statistic of those measures (eg, mean pain extent across a sample).

^b^Whether methods analyzed a cross section of a population (“population”), an individual over “time period”, a population over time (“both”), or pairs of manikin reports (“pairs”). Examples of pairs of manikin reports include a clinician and a patient each filling in a manikin to describe that patient’s pain or consecutive pairs of manikins from a set one patient filled in over time.

^c^Change over time only refers to direct analysis of manikin reports across a time period and does not include studies that compared individual-level manikin measures at different time points.

^d^Discriminant analysis is a statistical classification technique.

## Discussion

### Summary of Findings

This scoping review identified 92 studies that used digital pain manikins for data collection. Most studies were cross-sectional (64/92, 70%) clinical studies (51/92, 55%) using manikin-derived summary measures to answer a clinical question, although a large minority of studies (51/92, 55%) were methodological work on the development or validation of digital pain manikins. Most studies (84/92, 91%) used 2D or pseudo-3D manikins, and manikins were most commonly pixel based. We identified 10 pain constructs expressed by individual drawing–level summary measures, with significant methodological variation between summary measures for the same pain construct.

Pain extent was the most commonly measured pain construct. It does not make as much use of the available spatial information when compared to other measures such as widespreadness and symmetry. For example, a hypothetical manikin report where alternate pixels were reported as painful would have the same pain extent score as one where every pixel in the lower half of the body is marked as painful, but widespreadness measures would distinguish between these two pain drawings. All studies reporting pain location either used region-based manikins or overlaid predefined areas onto pixel-based manikins to calculate these measures.

While pain is sometimes reported as a single entity, it can be a complex system made up of different pathophysiological causes from different anatomical sites with different characteristics (eg, a constant ache from musculoskeletal pain and a shooting neuropathic pain). A small proportion of manikins captured location-specific pain quality, but most studies (62/92, 67%) only collected pain location. Asking people to report on pain in general may miss nuances regarding the specific pain components, but it has the advantage of avoiding the difficult challenge for patients in disentangling the sources of their pain, which may not be apparent to them.

Heat maps and location frequency were the most common multidrawing summary methods. Multidrawing analysis methods were less commonly used than summary methods and were mostly concerned with different ways to quantify the similarity between pairs of manikins (often for development or validation studies), the change over time in one person, or grouping similar manikins together.

There was a general lack of clarity around methodology in the literature, with many studies missing basic information such as whether a manikin was pixel or region based. It was often difficult to determine what construct was intended to be measured or whether two measures used equivalent methods. We observed many studies reporting the number they calculated for a summary measure without explaining the process in sufficient detail for it to be reproduced. The issue of missing information was present in the descriptions of the acquisition methods, as well as of the summary and analysis methods.

### Relation to Other Studies

We are not aware of any previous work that classified manikin-derived measures by pain construct, but 2 previous reviews looked at manikin-derived summary measures. The systematic review of digital pain manikin smartphone apps by Ali et al [11] found 9 manikin-derived summary measures, substantially fewer than the 31 found by our review. Although their review only included manikins available in app stores, all measures they reported were also found by our review. They suggested that there is a need to assess the measurement properties of smartphone-based pain manikins. Our mapping of pain constructs may help future work in this area as assessing the validity of measure requires an understanding of the construct it purports to measure.

One aspect of the systematic review of methodological milestones for the development of pain manikins by Shaballout et al [7] was manikin-derived measures, which they split into “simple measures” and “topographic measures.” They highlighted the need for standardization and the difficulty of comparing results between studies, advocating for the adoption of a common body template. They noted that digital pain manikins have the potential to record more pain attributes (eg, intensity or depth) when compared to paper manikins and that they expect further development in manikin-derived measures and analysis methods in future. Our study extends their work describing manikin-derived measures with an updated search that included more recent studies and a more detailed mapping of manikin-derived measures. On the basis of our findings, we concur with their suggestion that there is a need for standardization.

### Study Limitations

A limitation of our review is that we aimed to extract detailed data on methodological aspects of included studies, whereas manikin methods were often not our focus. Consequently, many of the included studies lacked detailed information on the extracted data items. However, including a broader range of studies made our review more comprehensive than if we had restricted it to those with a methodological focus.

Another limitation is that we restricted our search to published literature and did not include gray literature or apps and software not reported in literature, so we may have missed relevant measures and analysis methods from those sources. However, as all measures found by the app review by Ali et al [11] were also identified in our review, this suggests that we managed to identify a comprehensive set of manikin-derived pain constructs. Similarly, we restricted our search to digital pain manikins and excluded paper-based manikins. There is a possibility that by excluding paper manikins, we have missed summary measures or pain constructs that were not represented in the digital manikin set, but we believe that this had limited impact on our findings and conclusion.

Finally, we were unable to investigate measurement properties of manikin-derived summary measures as originally planned in our registered protocol because the high level of heterogeneity among methods of measuring constructs did not allow a meaningful synthesis. This review focused on identifying manikin-derived pain constructs (part 2 of our registered protocol’s objective 3). All remaining objectives were addressed elsewhere [103,104].

### Implications for Research

Manikin summary and analysis methods that make better use of location-specific pain aspects (such as location-specific pain intensity) should be developed. While 33 of the 92 included studies used manikins that captured location-specific pain aspects, only 12 (13%) of the reported individual-level summary measures, 4 (4%) of the multimanikin summary methods, and 0 (0%) of the multimanikin analysis methods used these data. For clinical studies with no plan to use location-specific pain aspects, the benefit of having these data available should be weighed against the additional burden to the patient in reporting them. This is in line with the consensus statement of recommendations to address respondent burden associated with patient-reported outcome assessment by Aiyegbusi et al [105], who also highlighted the need to consider the complexity and completion time of patient-reported outcome measures, which is particularly relevant when selecting a digital pain manikin for data collection.

Summary measures should be chosen with consideration of the underlying pain construct they attempt to measure and its relevance to the disease or clinical area being studied. Summary measures inherently lose spatial information, and very few studies used analysis methods that made use of this information. Different summary measures lose different parts of the spatial information; pain extent preserves the area but not the location of the pain, and widespreadness attempts to preserve an aspect of the location but not the area. This links to our previous recommendation to only capture location-specific information when there is a specific reason to do so. We recommend first identifying the pain constructs to be measured, then selecting appropriate summary measures for that construct, and finally selecting a pain manikin from which those summary measures can be derived with minimum participant burden. Through our review, it becomes evident that new methods may need to be developed to summarize multiple constructs simultaneously.

Our results suggest a need for standardization in pain manikin measures, whether this means settling on a single manikin or developing measures that are comparable between manikins. Due to the variety of methods and manikins used, even widely reported measures were not generally comparable between studies, meaning it would not be possible to assess measurement properties or validity across digital pain manikins as a whole. The lack of standardization may also lead to confusion in the context of clinical care. For example, a pain extent of 57% on one manikin is not necessarily the same as 57% on a different manikin, which could cause issues when translating results from research to clinical practice or when seeing patients who are using various different manikins. We contrast this with the standard methods of validating and developing questionnaires, where it is widely accepted that new questionnaires should not be developed if there is an existing validated questionnaire available. We recommend that researchers consider whether there is a suitable existing manikin before developing a new manikin, with future research focusing on providing insights into the practical benefits and limitations of different manikins as tools for various clinical and research applications (eg, the differences between the ability of 2D and 3D manikins to accurately capture pain in different contexts). Efforts should also be made to standardize the reporting of manikin studies and to agree on consistent terminology, ensuring terms such as “pain extent” and “widespreadness” are used consistently within the field; our review provides a strong foundation for this standardization.

We also suggest that it is not realistic that the field will settle on one manikin, particularly as different manikins may be appropriate for different research questions, and that efforts should be focused on developing methods to compare findings across manikins. One approach to this could be defining translations of multiple different manikins to one underlying representation, so that data collected on different manikins can then be summarized and analyzed in a consistent way. This is analogous to the problem of magnetic resonance imaging (MRI) dataset harmonization, where the specific machine used to collect MRI scans makes applying machine learning techniques across datasets challenging [106]. One approach to analyzing MRI scans is building a graph representation where the links between anatomical areas are an explicit part of the data format. A graph is a mathematical concept consisting of nodes that are linked by edges. For example, in a map of a social network, nodes would represent individual user accounts and edges would represent whether those users are connected. In a graph-based manikin, the nodes would represent individual anatomical locations and would be linked by edges only if they are anatomically adjacent. Future work could use a similar strategy of developing a graph-based manikin representation to solve the problem of standardization by defining translations of multiple manikins to the same graph representation. This would require a 2-way mapping between each manikin and the graph representation, allowing conversion between any manikins for which this mapping exists. To convert data collected from manikin A to manikin B, the data would be converted from manikin A to the graph-based representation and then from the graph-based representation to manikin B. Validation of this conversion would need to carefully account for the different shapes and sizes of different manikins to ensure that measures such as pain extent are preserved. A graph-based representation would also open the door to novel summary and analysis methods making use of the additional anatomical structure encoded in the format.

In addition to an evaluation of the practical benefits and limitations of different manikins as tools for various clinical and research applications, future work could also address questions that were outside the scope of this study, including an exploration of the potential applications of artificial intelligence, computer vision, and machine learning techniques in this domain.

### Conclusions

Our review identified a substantial number of studies that used digital pain manikins for data collection, with the majority reporting relatively simple measures and methods of summarizing pain drawings. Only a few studies went beyond summarizing to perform a direct analysis of the spatial data. The fact that information on pain location and other location-specific pain aspects (such as pain intensity or pain quality) collected through digital pain manikins was often not used in summary measures and methods suggests that the rich information available from pain drawings is currently not being fully harnessed. Future work should focus on developing more advanced summary and analysis methods that harness the spatial nature of pain drawings by better incorporating anatomical and clinical knowledge, while also improving reporting and standardization of pain constructs and methods through which they are measured. Together, this will contribute to expediting the use of digital manikins to support pain outcome measurement in both research and clinical care.

## Appendix

Multimedia Appendix 1 PRISMA-ScR checklist.

Multimedia Appendix 2 Full search strategy.

Multimedia Appendix 3 Full list of data extraction items.

Multimedia Appendix 4 Construct definitions.

Multimedia Appendix 5 Full data extraction spreadsheet.

## Abbreviations

CHOIR: Collaborative Health Outcomes Information Registry
MRI: magnetic resonance imaging
PRISMA: Preferred Reporting Items for Systematic Reviews and Meta-Analyses
PRISMA-ScR: Preferred Reporting Items for Systematic Reviews and Meta-Analyses Extension for Scoping Reviews

## Data Availability

All data generated or analyzed during this study are included in this published article and in Multimedia Appendix 5.
