# Supplementary material for: Summary and Analysis of Digital Pain Manikin Data in Adults With Pain Experience: Scoping Review
Source: J Med Internet Res. 2025 Aug 22;27:e69360. doi: 10.2196/69360 (PMC12413573; doi:10.2196/69360)
Supplement: Multimedia Appendix 2 [file jmir_v27i1e69360_app2.docx]

Multimedia Appendix 2 - full search strategy

Medline and Embase via Ovid online (22^nd^ August 2023)

1. exp MANIKINS/

2. exp Visible Human Projects/

3. exp Medical Illustration/

4. manikin*.tw.

5. mannequin*.tw.

6. (pain adj3 drawing*).tw.

7. (pain adj diagram*).tw.

8. (pain adj3 map*).tw.

9. (pain adj3 chart*).tw.

10. (body chart*).tw.

11. (body drawing*).tw.

12. (body map*).tw.

13. (body diagram*).tw.

14. 1 or 2 or 3 or 4 or 5 or 6 or 7 or 8 or 9 or 10 or 11 or 12 or 13

15. exp PAIN/

16. exp Pain Measurement/

17. exp Pain Management/mt [Methods]

18. pain*.tw.

19. 15 or 16 or 17 or 18

20. 14 and 19

21. animals/ not (humans/ and animals.mp.)

22. 20 not 21

23. limit 22 to english languageCINAHL Plus (3rd November, 2020; 378 titles)

1. MH manikin

2. MH visible human project

3. MH medical illustration

4. TX manikin*

5. TX mannequin*

6. TX pain adj3 drawing*

7. TX pain adj3 diagram*

8. TX pain adj3 map*

9. TX pain adj3 chart*

10.TX body chart*

11.TX body drawing*

12.TX body map*

13.TX body diagram*

14. 1 OR 2 OR 3 OR 4 OR 5 OR 6 OR 7 OR 8 OR 9 OR 10 OR 11 OR 12 OR 13

15. MH pain

16. MH pain management

17. MH pain measurement

18 TX pain

19. 15 OR 16 OR 17 OR 18

20. 14 AND 19

Scopus (22^nd^ August 2023)

( ( TITLE-ABS-KEY ( pain W/5 mannequin ) ) OR ( TITLE-ABS-

KEY ( pain W/5 manikin ) ) OR ( TITLE-ABS-KEY ( pain W/5 chart ) ) OR ( TITLE-ABS-

KEY ( pain W/5 drawing ) ) OR ( TITLE-ABS-KEY ( pain W/5 diagram ) ) OR ( TITLE-ABS-

KEY ( pain W/5 map ) ) ) AND NOT ( INDEX ( medline ) OR INDEX ( embase ) )

IEEE Xplore (22^nd^ August 2023) [Result of the Command Search that uses free text

and nested concepts; maximum 7 wildcards allowed per search]

(((“manikin*” OR “mannequin*” OR “pain NEAR drawing*” OR “pain NEAR map*” OR “pain NEAR

diagram*” OR “pain NEAR chart*” OR “body NEAR drawing” OR “body NEAR map” OR “body NEAR

diagram” OR “body NEAR chart”)))ACM Digital Library (3rd November, 2020; full text: 1095 titles]

“manikin*” OR “mannequin*” OR "pain drawing*" OR "pain map*" OR "pain diagram*" OR "pain

chart*" OR "body drawing*" OR "body map*" OR "body diagram*" OR "body chart*"
