# Supplementary material for: Summary and Analysis of Digital Pain Manikin Data in Adults With Pain Experience: Scoping Review
Source: J Med Internet Res. 2025 Aug 22;27:e69360. doi: 10.2196/69360 (PMC12413573; doi:10.2196/69360)
Supplement: Multimedia Appendix 3 [file jmir_v27i1e69360_app3.docx]

Multimedia Appendix 3 - full list of data extraction items

| Objective 1 |  |
| --- | --- |
| Study characteristics | Country  Publication year  Clinical purpose (e.g. aetiology, understanding pain prevalence/patterns in particular groups, diagnosis, identifying deterioration/change in disease status, assessing recovery/response to treatment)  Study aim (as reported by the authors)  Cross sectional or longitudinal |
| Setting and population characteristics | Pain type (induced/natural)  Clinical area (e.g. lower back pain, fibromyalgia)  Population description (e.g. healthy volunteers, people with arthritis)  Race/ethnicity  Number of participants included in the analysis  Age  Gender/sex |
| Objective 2a |  |
| Manikin characteristics | Name  Dimensions (e.g. 2d/3d)  Body area (e.g. whole body, head only)  Views (e.g. front, side)  Detail level (e.g. pixel, grid based, predefined areas)  Location-specific pain aspects (e.g. intensity, quality) |
| Individual manikin summary measures | Name  Description/definition of measure |
| Objective 2b |  |
| Methods | How is measure calculated (automated/manual/combination/not reported)*  Description of method (analysis methods/algorithm, assessment criteria)  Measure built into manikin (yes/no) |
| Objective 3 |  |
| Other analysis methods | Name  Description of method  Level of analysis (population, time period) |

*We defined “manual” methods to be any methods requiring human intervention on an individual manikin basis.
