# Supplementary material for: Summary and Analysis of Digital Pain Manikin Data in Adults With Pain Experience: Scoping Review
Source: J Med Internet Res. 2025 Aug 22;27:e69360. doi: 10.2196/69360 (PMC12413573; doi:10.2196/69360)
Supplement: Multimedia Appendix 4 [file jmir_v27i1e69360_app4.docx]

Multimedia Appendix 4 - construct definitions

| **Construct** | **Definition** |
| --- | --- |
| Pain extent (n=53) | The area of pain, regardless of the location |
| Location (n=28) | Quantification or description of the physical location of pain |
| Widespreadness (n=21) | The extent to which pain is present throughout the body rather than concentrated in a specific location |
| Pain quality (n=9) | The pain qualities included in a pain drawing, regardless of the location |
| Laterality (n=7) | Whether pain is present on one or both sides of the body split vertically |
| Shape (n=5) | Spatial measures of the area of pain such as the length, width, or product thereof |
| Location-specific intensity (n=4) | Weighted score for pain intensity using location-specific pain intensity information |
| Overlap (n=3) | The area of intersection of two distinct co-occurring sensations |
| Mismatch (n=3) | The area of non-intersection of two distinct co-occurring sensations |
